# Testing the effectiveness of an innovative information package on practitioner reported behaviour and beliefs: The UK Chiropractors, Osteopaths and Musculoskeletal Physiotherapists Low back pain ManagemENT (COMPLeMENT) trial [ISRCTN77245761]

**DOI:** 10.1186/1471-2474-6-41

**Published:** 2005-07-20

**Authors:** David W Evans, Nadine E Foster, Martin Underwood, Steven Vogel, Alan C Breen, Tamar Pincus

**Affiliations:** 1 School of Health and Rehabilitation, Keele University, Staffordshire, UK; 2 Primary Care Sciences Research Centre, Keele University, Staffordshire, UK; 3 Centre for General Practice and Primary Care, Barts and The London, London, UK; 4 Research Centre, The British School of Osteopathy, London, UK; 5 Institute for Musculoskeletal Research and Clinical Implementation, Bournemouth, UK; 6 Department of Psychology, Royal Holloway, University of London, London, UK

## Abstract

**Background:**

Low back pain (LBP) is a common and costly problem. Initiatives designed to assist practitioner and patient decisions about appropriate healthcare for LBP include printed evidence-based clinical guidelines. The three professional groups of chiropractic, osteopathy and musculoskeletal physiotherapy in the UK share common ground with their approaches to managing LBP and are amongst those targeted by LBP guidelines. Even so, many seem unaware that such guidelines exist. Furthermore, the behaviour of at least some of these practitioners differs from that recommended in these guidelines.

Few randomised controlled trials evaluating printed information as an intervention to change practitioner behaviour have utilised a no-intervention control. All these trials have used a cluster design and most have methodological flaws. None specifically focus upon practitioner behaviour towards LBP patients. Studies that have investigated other strategies to change practitioner behaviour with LBP patients have produced conflicting results. Although numerous LBP guidelines have been developed worldwide, there is a paucity of data on whether their dissemination actually changes practitioner behaviour. Primarily because of its low unit cost, sending printed information to large numbers of practitioners is an attractive dissemination and implementation strategy. The effect size of such a strategy, at an individual practitioner level, is likely to be small. However, if large numbers of practitioners are targeted, this strategy might achieve meaningful changes at a population level.

**Methods:**

The primary aim of this prospective, pragmatic randomised controlled trial is to test the short-term effectiveness (six-months following intervention) of a directly-posted information package on the reported clinical behaviour (primary outcome), attitudes and beliefs of UK chiropractors, osteopaths and musculoskeletal physiotherapists. We sought to randomly allocate a combined sample of 1,800 consenting practitioners to receive either the information package (intervention arm) or no information above that gained during normal practice (control arm). We collected questionnaire data at baseline and six-months post-intervention. The analysis of the primary outcome will assess between-arm differences of proportions of responses to questions on recommendations about activity, work and bed-rest, that fall within categories previously defined by an expert consensus exercise as either 'guideline-consistent' and 'guideline-inconsistent'.

## Background

Low back pain (LBP) is a common and costly problem [1,2]. As a result, the volume of research produced worldwide on the subject has been increasing steadily for several decades. This has allowed for the formulation of evidence-linked recommendations based on a process of evidence synthesis, expert consensus and summarising evidence in systematically developed statements [3]. Healthcare for acute LBP based on these recommendations can be expected to be safe, effective and more cost-effective than current usual care. Over 70% of patients managed in this way are likely to become pain-free, with a recurrence rate of less than 25% over the course of twelve months [4,5].

With the aim of assisting practitioner and patient decisions about appropriate healthcare for specific clinical circumstances [6], several initiatives have delivered recommendations about LBP to various stakeholder groups, including healthcare personnel, patients and the general public [7-15]. Amongst such initiatives, printed evidence-based clinical guidelines have been developed for primary care and occupational settings, in several countries [16-19].

In the UK, the National Health Service (NHS) Executive commissioned the Effective Clinical Practice Programme of the Royal College of General Practitioners (RCGP) to develop evidence-based clinical guidelines for the management of acute LBP in primary care. These were developed and printed for the use of first contact primary care practitioners, including the professional groups of chiropractic, osteopathy and musculoskeletal physiotherapy in the UK. They were first published in 1996 [20], then revised and updated in 1999 [21]. Since then, the Faculty of Occupational Medicine of the Royal College of Physicians commissioned the development of equivalent multi-disciplinary guidelines for the occupational setting [22,23].

Chiropractors, osteopaths and musculoskeletal physiotherapists in the UK share some common ground with their approaches to managing LBP [24]. As a result, these professions have been considered to be a collective stakeholder group [1,20,21]. Together, they are involved in the management of approximately one-fifth of all cases of LBP in the UK [25,26], making them an important provider of care. Indeed, Maniadakis and Gray [2] estimated that in 1998, the annual combined direct healthcare costs for LBP delivered by these professions was £493 million (US$820 million, based on 1998 exchange rates).

The majority of these practitioners work in private practice, rather than within the NHS [15]. As a result, they are excluded from both the constraints [27-32] and potential support [8,33] offered by working within large organisations of healthcare delivery. Unlike general practitioners [34], few UK guidelines are aimed at chiropractors, osteopaths and musculoskeletal physiotherapists. With LBP accounting for more than half of their workload [15,35-40], recommendations for optimal LBP management should be relevant to these practitioners. Whilst anecdote and some empirical data suggest that many of these practitioners are naïve to the existence of LBP guidelines, they do express a desire for a clear rationale in their LBP clinical decision-making [41]. Several studies show that the clinical behaviour of members of these professional groups diverges from guideline recommendations [38-40,42] and that this may relate to the beliefs of these practitioners [43-49]. It is therefore timely to explore ways to inform and optimise aspects of their clinical practice.

### Evidence for interventions to change practitioner behaviour

Healthcare research findings are usually published in peer-reviewed scientific journals. However, this neither ensures that these findings reach practitioners (dissemination) nor that they are used in routine clinical practice (implementation) [50-54]. Dissemination involves raising awareness of research messages whereas implementation involves getting the findings of research adopted into practice [55].

Interventions that aim to change the behaviour of healthcare practitioners essentially fall into two groups: those that use broadly educational approaches and those that use financial or organisational prompts [56]. There is a diverse array of educational interventions [57,58]. 'Active' educational interventions, with which practitioners are directly engaged, are usually regarded as more effective at changing behaviour than 'passive' interventions, which act as vehicles for disseminating information such as behaviour recommendations, and are commonly in a written format [59-62]. Multifaceted interventions are widely thought to be more effective than single interventions, even though empirical evidence does not fully support this opinion [63].

The most recent and thorough systematic review of the evidence for printed information as an intervention to change practitioner behaviour [61] included several randomised controlled trials (RCTs) with a no-intervention control [64-70]. These studies reported mixed results. Notably, all were of a cluster design, with the unit of randomisation for intervention allocation being groups of practitioners, such as regions, practices or clinical teams. Cluster designs have potentially limited external validity since the results may not be applicable to other locations and settings. Most of these RCTs suffered from methodological flaws, with their study designs and analyses failing to allow for effects of clustering [71,72]. Similar methodological problems were identified in a further cluster RCT [73], published after the search period of the review. Of all these RCTs, only one [67] included practitioner behaviour towards LBP patients, however, the intervention was poorly described. In addition, none of these RCTs focused on members of the chiropractic, osteopathy and musculoskeletal physiotherapy professional groups in the UK.

Given the methodological problems associated with cluster RCTs [74-76], it is surprising that they dominate the scientific literature on changing practitioner behaviour with printed guidelines. Indeed, one large RCT that recently attempted to assess guideline implementation using a cluster design had to abandon this comparison because of methodological problems [77]. Furthermore, the practical ease of posting written educational material to individual practitioners, in contrast to more 'active' interventions, should lend itself to a non-cluster RCT. The effectiveness of disseminating printed information on practitioner behaviour is therefore yet to be tested for independent effects on individual practitioners in a RCT. In addition, the impact of printed guidelines on the management of LBP has not been adequately assessed.

To date, studies investigating strategies to change practitioner behaviour towards LBP patients have produced conflicting results. Consistent with studies from other healthcare topics, a cluster RCT by Bekkering et al [78] found a statistically significant positive change in some of the targeted behaviours of Dutch physiotherapists using a multifaceted educational intervention, compared to passive dissemination of printed guidelines alone. The multifaceted intervention consisted of mostly active components, utilising education, discussion, role-playing, feedback and reminders. In another cluster RCT, the same research group produced small changes in some of the targeted behaviours of Dutch GPs [80]. In this study, the multifaceted intervention consisted of active and passive components, including the Dutch LBP guideline for GPs, the LBP guideline for occupational physicians, a 2-hour educational and clinical practice workshop, 2 scientific articles on LBP management, a tool for patient education and a tool for reaching agreement on LBP care with physical, exercise, and manual therapists. In a similar manner, yet another cluster RCT [81] found a small but statistically significant positive behaviour change in groups of US primary care physicians, following an active educational intervention based on an education/audit/feedback model with local peer opinion leaders. Conversely, a cluster RCT in a sample of UK general practitioners showed that an active, tailored, multifaceted educational intervention, including the identification and targeting of specific barriers to behaviour change via outreach visits, failed to demonstrate a statistically significant change in prescribing or referral behaviour [82]. The variability of these results may be partly explained by contextual factors, such as the specific behaviour(s) targeted, the professional group of the practitioners, and the healthcare system and practice setting in which the practitioners work.

Given the expense of active interventions, the comparative low unit cost of sending printed information to large numbers of practitioners makes it an attractive dissemination and implementation strategy. It may be seen as more efficient to adopt a less effective but less costly implementation strategy [61,63,83]. Whilst the effect size of passively disseminated printed information is likely to be small at an individual practitioner level, the potential of the strategy relies on the capacity for small behaviour changes in large numbers of practitioners to have an overall substantive impact upon a healthcare system [84]. Even quite small changes at an individual practitioner level could translate, at a population level, into a worthwhile change in the management of large numbers of patients. This notion may have particular merit for an economically costly healthcare issue such as LBP [2]. Nevertheless, the evident international popularity of printed guidelines for the management of LBP [16-19] ought to be matched by rigorous evaluation through systematic research [52].

We have designed a prospective, pragmatic RCT for a large number of practitioners working predominantly in primary care settings. The primary aim of the study is to test the short-term effectiveness (six-months following intervention) of a directly-posted information package on the reported behaviour and beliefs of a combined sample of chiropractors, osteopaths and musculoskeletal physiotherapists in the UK. We are not aware of any previous implementation research that has utilised a randomly allocated no-intervention control, in which the individual practitioner was the unit of randomisation. Additionally, to our knowledge no research has investigated these issues across these three professional groups.

Our secondary aims are:

i) to describe patterns of reported behaviour, attitudes and beliefs, within and between professional groups at baseline,

ii) to identify baseline factors that are associated with a change in reported behaviour of these practitioners (such as demographic data, attitudes and beliefs of practitioners),

iii) to make recommendations for future research on implementation strategies in these professional groups.

## Methods

### Trial design

This prospective, pragmatic, two-arm RCT was conducted with practitioners from the three groups of chiropractic, osteopathy and musculoskeletal physiotherapy in the UK. The unit of randomisation and analysis throughout the study was the individual practitioner. This allowed for a nationally representative, non-clustered study population. Participants from each professional group were randomly allocated to receive either a directly-posted information package (intervention arm) or no information over and above that gained during normal practice (control arm). Ethical approval was obtained from the London Multi-centre Research Ethics Committee. We collected data using self-completed questionnaires at baseline and six-months post-intervention. Questionnaire content, format and response rates were tested in a pilot survey (n = 150).

### Study population

**Figure 1:**
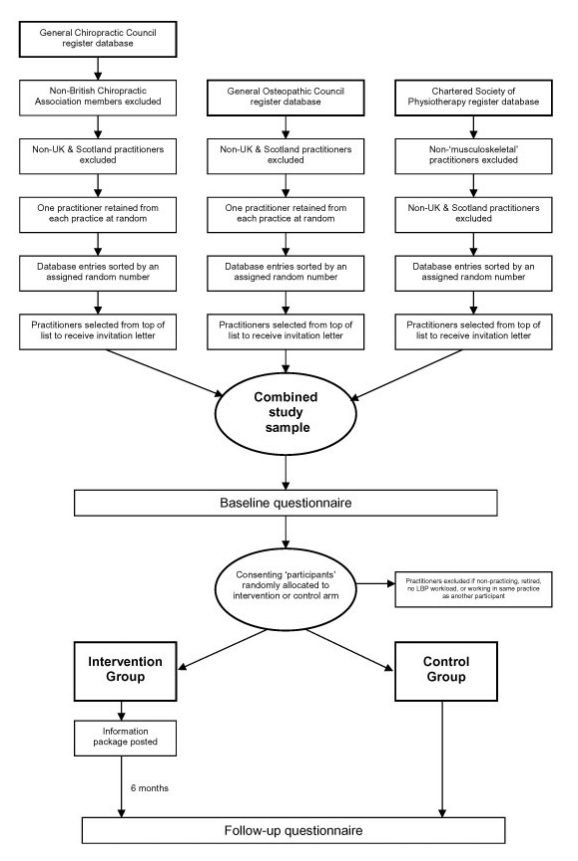
Trial design

Participants were members of the professional groups of chiropractic, osteopathy and musculoskeletal physiotherapy, working in the UK and predominantly in a primary care setting. A substantial majority of chiropractors and osteopaths work exclusively in the private sector. Musculoskeletal physiotherapists work in both private and NHS sectors. Based on the pilot study response rates for each of the three professional groups, roughly proportional random samples were drawn from the respective professional register of each professional group (Table 1). Each selected practitioner was then sent an invitation to take part in the study (Figure 1). The study began in November 2003. Invitation letters, study information sheets and baseline questionnaires were sent together, with one reminder including further copies of all documents for non-responders. Only completed baseline questionnaires received prior to our predetermined baseline cut-off date (1^st ^March 2004) were included. Following this, the intervention package was posted. Follow-up questionnaires were posted six-months following intervention. Non-responders to the follow-up questionnaire were also sent a reminder. The eligibility (and exclusion) criteria are in Table 1.

**Table 1:** Sample eligibility and recruitment

**Professional group**	**Eligibility criteria**	**Total population meeting criteria**	Available number in population following initial exclusions*	**Number randomly selected (% of total population)**
***Chiropractors***	Registered with the General Chiropractic Council *and *the British Chiropractic Association	1071 (100%)	611	611 (57.0%)
				
***Osteopaths***	Registered with the General Osteopathic Council	2718 (100%)	1868	1368 (50.3%)
				
***Physiotherapists***	Registered with the Chartered Society of Physiotherapy *and *self-selected as 'musculoskeletal' by speciality	3586** (100%)	3230	1625 (45.3%)
**Total =**		**7375 (100%)**	**5709**	**3604 (48.9%)**

* Practitioners with addresses outside of the UK and in Scotland were excluded prior to posting invitations, and only one practitioner from each practice was eligible, being randomly selected from each address to avoid contamination (chiropractors and osteopaths only).

** At the time of sampling, there were approximately 30,000 physiotherapists in the UK, of which only 3587 were listed by the Chartered Society of Physiotherapy as 'musculoskeletal' by speciality.

### Chiropractors

Following the Chiropractors Act (1994), the legal definition of a chiropractor in the UK is a registrant with the General Chiropractic Council (GCC). McTimoney chiropractors are a minority group within the profession, comprising approximately one third of these registrants. McTimoney chiropractors are known to practice in substantially different ways compared to other chiropractors [85]. For this reason, they have previously been excluded from another multidisciplinary study [24,86]. Therefore, in an attempt to increase the homogeneity of the chiropractors in this study, only British Chiropractic Association (BCA) members, who are not McTimoney trained, were included (i.e. non-BCA members were excluded). With relevant permissions, the GCC register was cross referenced with the BCA register to provide the chiropractic sampling frame in this study. This information was less than twelve months old at the time of recruitment (November 2003).

### Osteopaths

Following the Osteopaths Act (1993), the legal definition of an osteopath in the UK is a registrant with the General Osteopathic Council (GOsC). Whilst there is known to be considerable variation in practice within the profession [42], there are no established minority groups comparable to McTimoney chiropractors that needed excluding. Therefore, with permission, the GOsC register was used as the osteopathy sampling frame in this study. This information was less than twelve months old at the time of recruitment.

### Musculoskeletal physiotherapists

Almost all of the approximately 35,000 physiotherapists in the UK are registered with the Chartered Society of Physiotherapy (CSP). Within the register of the CSP at the time of sampling, there were self-selected clinical speciality groups, one of which was 'musculoskeletal'. In an attempt to increase the likelihood that contacted physiotherapists were directly involved in managing LBP patients, this subgroup of the CSP register was used, with permission, as the physiotherapy sampling frame in this study. This information was the best available at the time of recruitment, although it was approximately four years old.

### Exclusion criteria

Non-UK practitioners were excluded from the study by removing them from the professional register database sampling frames before the study sample was randomly selected. Likewise, all practitioners living or working in Scotland were excluded due to the likely confounding effects of the simultaneous, ongoing national multifactorial educational campaign, Working Backs Scotland [15,87].

Where possible, one practitioner was randomly selected from each practice prior to randomly drawing the study sample. Practice addresses were only available from the GCC and GOsC registers. The CSP register provided mostly home addresses and thus did not allow for the exclusion of practitioners working from the same practice. Inspection of chiropractic and osteopathy registers also indicated that many practitioners worked at more than one practice. Therefore, to reduce possible contamination, we excluded any practitioner (regardless of professional group) if the preferred contact address given on the baseline questionnaire was identical to that of a practitioner already recruited to the study.

Further exclusions were made from information given on returned baseline questionnaires. Practitioners who had retired, who were non-practicing, or who reported that they were not currently involved directly in the management of LBP patients were excluded (Figure 1).

### Participant recruitment

We drew a random sample from the respective professional registers of each professional group. Random selection from the electronic register databases was performed by allocating a randomly generated number in a computer spreadsheet (MS Excel 2002) to each practitioner in their respective register and then sorting the list by this number. The sample was drawn from the descending list as per the order of the sorted random numbers (Figure 1). The size of each sample drawn was approximately proportional to the size of the group in the UK at the time of recruitment (Table 1).

Each practitioner selected was sent, via 'recorded delivery' (signature required) post, a letter of invitation to take part in the study, a brief study information sheet based on the guidance from the Multi-Centre Research Ethics Committee, a pre-paid reply envelope and the baseline questionnaire. A removable consent form was incorporated in the baseline questionnaire, which also gave participants opportunity to provide a preferred contact address. Only those respondents providing written consent to participate were included in the study.

We calculated response and recruitment rates from an 'adjusted sample' that excluded undelivered questionnaires and those returned to the research team (e.g. 'addressee gone away') plus those respondents who indicated that they were retired, no longer in clinical practice, or whose workload did not include LBP patients. As expected, the older physiotherapy register database provided more exclusions in this way than the other register databases. Practitioners not responding to the first posting of these documents were sent a second and final copy of each after eight weeks, via normal post.

'Non-responders' were those where no response was received to either posting of the baseline questionnaire. In total, 20% of the non-responders from each professional group were followed up with a shortened version of the questionnaire, in order to collect demographic information and to explore potential selection bias.

### Consent and participant anonymity

Each practitioner selected from their professional register was allocated a unique study number prior to the posting of the baseline questionnaires. This number appeared on both consent and data collection sections of the baseline questionnaire, and allowed for the efficient tracking of both responders and non-responders throughout the study.

On receipt of completed baseline questionnaires, one member of the research team removed the consent section from the accompanying data collection section and stored them separately. Thus, the unique study number became the only source of identification on each questionnaire. This ensured that participant confidentially was maintained, and ensured that the study team were blind to intervention allocation. Only one member of the research team, not involved in data entry or intervention allocation, had access to a password-protected database on a secure computer containing the personal identifying information associated with each unique study number.

### Randomisation and intervention allocation

The baseline questionnaire was completed prior to subsequent intervention allocation (Figure 1). Practitioners that responded to the baseline questionnaire and consented to take part further in the study became the study 'participants'. A member of the research team, based in another institution and who was not involved in the data collection, produced a blind, random allocation of each participant to either control or intervention arms, using only a list of participant's anonymous unique study numbers in StatsDirect software package. The intervention packages were posted with a brief covering letter to those allocated to the intervention arm in March 2004.

### Interventions

#### Information package

The intervention consisted of an innovative information package, posted directly to participants. The package clearly details the evidence-based management of acute back pain, based on recommendations from the UK evidence-based and evidence-linked multi-disciplinary primary care [21] and occupational [22] LBP guidelines. We originally considered producing our own information package. However, when designing the study we identified an existing, high quality, package that met our needs. This package was originally developed as part of an ongoing national, multi-intervention educational campaign in Scotland led by the Health Education Board of Scotland and the Health and Safety Executive, entitled 'Working Backs Scotland' (WBS) [15]. We used exactly the same package as the WBS package with an additional covering letter, which explained the origins of the package. We did not have sufficient resources to produce a new version of the pack to the same quality, in order to remove its Scottish origins. Since the issues in the management of LBP are likely to be similar in all parts of the UK, this should not affect the appropriateness of the package for our current purpose.

The novel approach taken by the WBS initiative was to produce a comprehensive and cohesive information package, suitable for multiple stakeholders (summarised in Table 2), as part of a national initiative similar to a high-profile and largely successful Australian study [9,10,88].

**Table 2:** Components of the 'Working Backs Scotland' information package

**Targeted stakeholder**	**Component of package**
Patients	The Back Book (1st edition)
	Health & Safety Executive: Back in Work booklet
	Contextualised A4 sheet with recommendations
	Poster with recommendations for patients
	
All providers	Introduction letter from Working Backs Scotland
	
***Therapy Providers***	Contextualised A4 sheet with recommendations
	Royal College of General Practitioners: A4 guideline summary pamphlet
	Yellow Flags guideline summary
	
***General Practitioners***	Contextualised A4 sheet with recommendations
	Royal College of General Practitioners: A4 guideline summary pamphlet
	Yellow Flags guideline summary
	
***Pharmacists***	Contextualised A4 sheet with recommendations
	
***Employers***	Contextualised A4 sheet with recommendations
	Health & Safety Executive: Back in Work booklet

In order to maximise the likely success of the package, the contents were deliberately designed to target previously identified contextual 'barriers' to behaviour change. One suggested strategy for increasing the uptake of guideline recommendations has been local adaptation, in order to provide a sense of relevance and local 'ownership' of the recommendations [54,89,90]. However, there is some evidence that local adaptation of national guidelines has no effect on practitioner behaviour [91], suggesting that relevance and ownership should be provided in some other way. One alternative for providing these has been the production of unidisciplinary guidelines. An example of this has been the recent formation of physiotherapy guidelines in the Netherlands [13], an equivalent of which is also currently under development in the UK [92]. Unidisciplinary guidelines have the further advantage of being able to provide best practice recommendations on technical areas of clinical practice that have little relevance outside a particular healthcare professional group.

In the WBS package, an attempt has been made to simultaneously provide relevance for all stakeholder groups by supplying each with an individual, tailored A4 recommendation sheet, every sheet being consistent with the others [87]. Each A4 sheet includes only those recommendations most likely to be relevant to that particular group. This allows every stakeholder to see the consistent messages provided by the recommendations, with the aim of reinforcing these messages. Importantly, the specific behaviours targeted for all stakeholder groups are kept to a minimum [84] and focus predominantly upon LBP patients staying active, avoiding bed-rest, and staying at or returning to work. Intended specifically for the healthcare practitioners is a copy of the brief summary A4 pamphlet version of the RCGP guidelines [21], and an A4 summary sheet explaining the concept of psychosocial 'Yellow Flags' [93]. The package also includes a copy of the patient-orientated advice booklet, the first edition of 'The Back Book' [94], which has been formally tested by Burton et al [95], and a leaflet designed for employers and workers, 'Back in Work' [96], the messages of which are consistent with the guidelines.

Much like the principal recommendations of the original sources on which the WBS package was based [21-23], the wording of the recommendations on each contextualised A4 sheet are clearly written in the form of behavioural instructions; an important attribute in any intervention designed to change practitioner behaviour [97,98]. Credibility of the package is reinforced by several declarations that the contents of the package are based on the best international research evidence [97]. Furthermore, the commissioning body was expected to be well respected by all stakeholders (Waddell G, personal communication). The issue of perceived ownership of the material [99] was addressed through considerable effort in enlisting and displaying the support of a wide range of local and national professional groups and societies (22 in total). These groups, including bodies representing the chiropractic, osteopathy and physiotherapy professions, endorsed both the initiative and the information package.

#### Control arm

Participants in the control arm were sent the standard brief description of the study with the baseline questionnaire, but were sent no further information prior to the six-months post-intervention follow-up questionnaire. Therefore, they received nothing other than they would during usual clinical practice. Of course no control could, nor should, have been exerted over other sources of information available to either arm of the trial. Being a pragmatic RCT with a large sample size, any other influences on clinical practice are likely to have been equal for each arm.

### Questionnaire development

The choice of outcome measures was guided by the findings from previous qualitative studies [41,44]. Permission to use previously published material was gained from the original authors of each outcome measure. The contents of the baseline and sixmonths post-intervention follow-up questionnaires are summarised in Table 3. The two most important outcomes measured in the study were (1) reported clinical behaviour (primary outcome), and (2) practitioners' attitudes and beliefs. Each outcome measure was slightly modified from the original version for the purposes of this study. A description and justification for each modification is given below.

**Table 3:** Information collected in the self-completed questionnaires

**Information**	**Measure**	**Original authors**	**Items**	**Baseline**	**6 months**
Demographic details	Age Professional group Years qualified NHS/Private Back pain workload Personal history of back pain	-	-	Yes	No
Reported behaviour relating to a patient vignette with non-specific acute low back pain and no 'red flags'.	Questions relating to a low back pain patient vignette (modified from original studies)	Modified from: Bombardier et al 1995 [105] Rainville et al 2000 [109] Buchbinder et al 2001 [10] Bishop & Foster 2005 [49]	3	Yes	Yes
Practitioner beliefs concerning the way that low back pain is likely to affect function	Modified Health Care Providers Pain and Impairment Relationship scale (HC-PAIRS)	Modified from: Rainville et al 1995 [110] Houben et al 2004 [46]	13	Yes	Yes
Practitioner self- confidence in managing patients with low back pain	Practitioner self-confidence scale	Modified from: Bush et al 1993 [117] Smucker et al 1998 [118]	4	Yes	Yes
Practitioner attitudes towards the use of research and evidence in practice	Practitioners connections with research Authority of information sources to influence practice	Modified from: Connolly et al 2001 [119]	12	Yes	Yes

### Outcome measure 1: Reported behaviour

**Figure 2:**
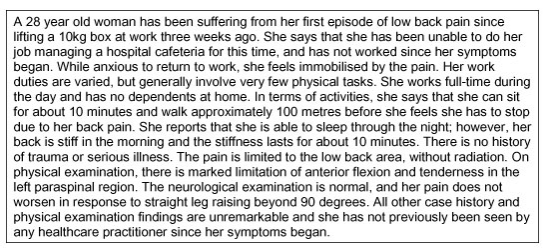
Patient vignette with non-specific acute low back pain and no 'red flags'.

The primary outcome measure was a measure of reported clinical practice behaviour, captured using questions related to a single vignette of a patient with non-specific acute LBP and no 'red flags' for possible serious spinal pathology (Figure 2).

Vignettes are hypothetical clinical presentations of patients intended to elicit from practitioners an underlying practice pattern or 'medical signature' [100]. Research has been conducted on the discordance between what physicians report they would do in response to a patient vignette and what they actually do in practice. Some investigators have raised concern to the value and validity of vignettes as an outcome measure in assessing practitioner behaviour [100-103]. Specifically, critics have argued that practitioner responses to vignette are likely to report what they perceive should be done rather than what they would actually do. However, a large, prospective study [104] has shown that vignettes are not only reliable, stable and valid measures of practitioner behaviour, but that they are more accurate than patient case notes (chart abstraction), which are often used as a measure of practitioner behaviour. Thus, despite understandable concerns of social desirability or acquiescence bias, changing responses towards vignettes may be considered a single, valid point along a causal pathway towards actual behaviour change.

We chose to use a vignette in the present study as we wanted to assess the practitioners' decisions about appropriate healthcare for a specific clinical scenario, which is precisely what clinical guidelines are intended to facilitate [6]. The vignette used was originally developed by Bombardier et al [105], and has since been used in several studies [10,106-108]. Some details of the vignette were anglicised and clarified prior to use, and further details regarding occupational duties and domestic responsibilities were added following comments received during the pilot study.

**Figure 3:**
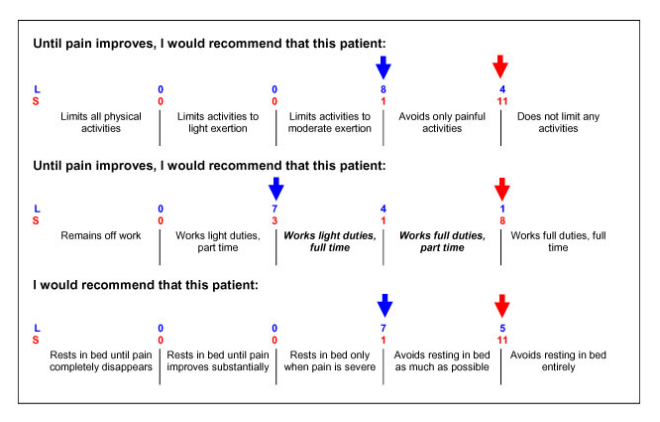
**Questions and corresponding response options relating to a low back pain patient vignette **The points of dichotomisation for each scale, based on expert consensus, are shown (with arrows). For each scale, the response option(s) to the right of each arrow were interpreted as consistent with guideline recommendations. The numbers above and between response options represent the frequency of expert opinion for the point of dichotomy relating to that scale. Lenient interpretations (L) are placed on the top row above response options, and strict interpretations (S) on the bottom row. The five-point scales are considered ordinal. However, two items of the scale related to Work will be combined (highlighted in bold italics) to form a four-point scale in this case.

In light of key recommendations consistent across guidelines from many countries [16-19], and in a manner similar to the work of Rainville et al [109] with US physicians and Bishop and Foster [49] with UK physiotherapists, the primary questions relating to the vignette were designed to explore practitioner recommendations for patient behaviour in relation to (i) Work, (ii) Activity, and (iii) Bed-rest. Other secondary questions were added to explore investigations (including x-rays) and initial treatment interventions (e.g. advice, manual therapy, electrotherapy and exercise). The format chosen to capture responses to each of these primary questions was a 5-point Likert-type scale (Figure 3), based on questions originally used by Rainville et al [109].

In order to inform the analysis of the results, and as the responses to these questions were being interpreted in light of guideline recommendations, an expert consensus exercise was performed. A group of 12 experts with extensive prior experience in LBP research were contacted independently and provided with a copy of the vignette and questions. Feedback from the experts confirmed that the five-point scales could be considered ordinal. However, it was recommended that two items of the scale related to Work should be combined (highlighted in bold italics in Figure 3) to form a four-point scale in this case.

There are two research questions relating to the primary outcome of reported behaviour. Each of these requires a different analysis to test for a statistically significant change between the two arms of the trial at the six-month post-intervention time-point. The two research questions are:

1. For each scale, can the intervention change the response proportions of 'guideline-consistent' and 'guideline-inconsistent' reported behaviour?

2. For each scale, can the intervention change the response proportions of reported behaviour across the entire range of responses?

The first of these questions represents the primary outcome measure for this study. It relates to changes in the proportions of responses either side of a predetermined cut-off point for each reported behaviour research question. Therefore, each of the original five-point scales will be dichotomised to create a new variable with categories defined as 'guideline-consistent' and 'guideline-inconsistent'. The location of the cut-off points for each scale is based on the expert consensus exercise. All experts were asked to give, where possible their 'lenient' and 'strict' interpretation of internationally agreed recommendations from primary care and occupational evidence-based guidelines [16-19] for each scale. These two interpretations will allow for a sensitivity analysis.

The wording and points of dichotomisation for each scale question are shown (with arrows) in Figure 3. For each scale, the response option(s) to the right of the arrow were interpreted as recommendations consistent with those of the guidelines. The positions of the red arrows indicate the location of cut-off points for the most frequent 'strict' expert interpretation of the guideline recommendations. The blue arrows indicate the location of cut-off points for the most frequent 'lenient' expert interpretation.

### Outcome measure 2: Practitioners' beliefs

Practitioners' beliefs regarding the relationship between back pain and physical function were measured using a modified version of the 13-item Health Care Providers Pain and Impairment Relationship Scale (HC-PAIRS) (Table 4), which was originally developed and validated by Rainville et al [110], and further studied by Houben et al [46]. The, responses for each item were collected with a 7-point Likert scale anchored from 'Completely disagree' (1) to 'Completely agree' (7). The scores are summed to form a total HC-PAIRS score, giving a theoretical range of possible scores from 13 to 91. Items 1, 6 and 12 were positively worded and were therefore reverse-scored for scoring and subsequent analysis. Factor analysis has shown the HC-PAIRS to measure a single factor of beliefs concerning the way in which LBP affects physical function [46]. Higher scores on the HC-PAIRS indicate a stronger belief that pain should affect daily function.

**Table 4:** Modified items of the HC-PAIRS. Modified components are highlighted in bold.

**Item No.**	**Item**
1	**Low **back pain patients can still be expected to fulfil work and family responsibilities despite pain
2	An increase in pain is an indicator that a **low **back pain patient should stop what they are doing until the pain decreases
3	**Low **back pain patients cannot go about normal life activities when they are in pain
4	If their pain would go away, **low **back pain patients would be every bit as active as they used to be
5	**Low **back pain patients should have the same benefits as the handicapped* because of their **painful **problem
6	**Low **back pain patients owe it to themselves and those around them to perform their usual activities even when their pain is bad
7	Most people expect too much of **low **back pain patients, given their pain
8	**Low **back pain patients have to be careful not to do anything that might make their pain worse
9	As long as they are in pain, **low **back pain patients will never be able to live as well as they did before
10	**Low **back pain patients have to accept that they are disabled persons, due to their pain
11	There is no way that **low **back pain patients can return to do the things that they used to unless they first find a cure for their pain
12	Even though their pain is always there, **low **back pain patients often don't notice it at all when they are keeping themselves busy
13	All of **low **back pain patients' problems would be solved if their pain would go away

* The following caveat to item 5 was added as a footer in the modified version: We are aware that the term 'handicapped' is not liked by many people who have completed this questionnaire in the past but the research team are unfortunately unable to change this previously validated questionnaire as we may affect the results if we did so.

The original version of the HC-PAIRS was intended to assess health care providers' attitudes and beliefs about the degree to which chronic LBP justifies impairments and disability. It was published in 1995, and was itself adapted from a questionnaire developed for chronic pain patients, the Pain and Impairment Relationship Scale (PAIRS) that was first published in 1988 [111].

Modifications were made to the original HC-PAIRS in the present study for several reasons. Firstly, the evidence-based primary care [21] and occupational [22] guidelines, upon which the intervention package was based, were specifically developed for the management of *low *back pain rather than 'back pain' per se. Furthermore, the primary care guideline in particular is specifically based on the evidence for *acute *LBP and is clearly labelled as such. Moreover, the term 'chronic back pain patients' that Rainville et al [110] used to modify the PAIRS scale was based on the premise that the attitudes and beliefs measured would be valid only in chronic pain patients. Thus, the assumptions behind the design of these scales were representative of the knowledge at the time of publication. Certainly, one major development in LBP research since the late 1980's and early 1990's is that psychosocial factors have been shown to be involved much earlier in the course of LBP than was originally thought. Therefore, even though the PAIRS has not been formally tested in any other population than chronic pain patients [111-114], it is reasonable to argue that the practitioner beliefs measured by the modified version of the HC-PAIRS will be appropriate in the context of acute LBP patients.

### Sample size calculation

The primary outcome measure for this trial was the proportion of 'guideline-consistent' and 'guideline-inconsistent' reported behaviour, relating to the patient vignette for the key variables of work, activity, and bed-rest (Figure 3). This analysis is also in need of more statistical power than the alternative analysis using change in responses across the whole scale. It is therefore the appropriate criterion by which to estimate the target sample size for the trial. In light of previous studies on practitioner behaviour change [59,73], we defined a change of 10% in reported behaviour between the proportions of practitioners from baseline to the six-months post-intervention follow-up as a clinically meaningful change. Thisdifference is the same as the median effect size reported in a review of guideline implementation studies reported in a subsequent review [61,63].

There are no existing data on 'guideline consistent' responses on which we could base our sample size calculation. To show a 10% difference in the proportion of these practitioners who, at the six-month post-intervention follow up, would give 'guideline consistent' responses to this questionnaire, the greatest statistical power is needed when proportions in each group are 45% and 55%. To show this difference at the 5% significance level with 90% power, valid data from at least 543 participants in each arm of the trial are needed for analysis (1086 total). Previous studies using a similar questionnaire format to measure outcomes have shown a response rate of between 31% and 65% [10,41,73,109,115]. Based on these figures, and the overall response rate of the pilot study, a recruitment rate of approximately 50% was anticipated at baseline. Allowing for 40% loss to follow-up, 900 practitioners had to be recruited into each arm of the trial at baseline (1800 total). Further allowing for the estimated recruitment rate of 50% at baseline, approximately 3600 practitioners needed to be invited to participate at the start of the study. A total of 3604 were actually invited. Such over-sampling strategies are typical of studies on healthcare practitioners based on survey methodology [e.g. [41,115]].

### Data handling and analyses

The anonymised questionnaire data have been transferred into electronic format using a pre-coded datafile via Statistical Package for Social Scientists (SPSS) Data Entry (version 3.0) software, which is designed to reduce systematic errors during data entry. Data were independently entered by an experienced data entry administrator, who was blind to the participant identities. For purposes of reducing error, 20% of all data entered in the SPSS datafile was checked by one of the research team, blind to participant intervention allocation at the time.

As this is a pragmatic trial, the primary analysis will be performed on an intention to treat basis, as recommended by Bland [71]. It will include all participants randomised, even if they did not subsequently receive or read the intervention package when allocated. A proposed secondary analysis will focus on participants who appear to have read the information package, as identified by means of responses to a question on the follow-up questionnaire acting as a proxy. Data will be analysed using SPSS (version 11.5). All significance tests will be two-sided and statistical significance will be set at the 5% level.

The reported behaviour outcomes require a different analysis for each of the two research questions. The dichotomous variable, with categories defined as either 'guideline-consistent' or 'guidelineinconsistent', will be analysed using a binomial regression model (primary analysis). This will allow for between-arm differences of the proportions of responses that fall within each category at follow-up to be tested. The points of dichotomy will be based on the 'lenient interpretation' for each variable, as per the expert consensus exercise (Figure 3). The 'strict interpretation' for each variable will be used to provide sensitivity analyses. The original ordinal data will be analysed using an ordinal regression model, in which between-arm differences of proportions across all follow-up reported behaviour data may be tested. Baseline reported behaviour responses will be included in each model as a covariate. The secondary clinical and demographic data collected at baseline will be inspected and will also be included as covariates in the analyses, depending on the proportions of variance shared between these variables.

## Conclusion

The COMPLeMENT trial is a prospective, pragmatic RCT of printed, evidence-based educational material that incorporates a no-intervention control. We have presented the rationale, design, and strategy for implementation of the trial, which incorporates a large number of practitioners working predominantly in a primary care setting. The primary objective of this study is to test the short-term effectiveness (six-months following intervention) of a directly-posted information package, that contains contextualised evidence-based recommendations for the management of acute back pain, on the reported behaviour and beliefs of a combined sample of chiropractors, osteopaths and musculoskeletal physiotherapists in the UK. The secondary objectives are to describe patterns of reported behaviour, attitudes and beliefs, within and between professional groups at baseline; to identify baseline factors that are associated with a change in reported behaviour of these practitioners; and to make recommendations for future research on implementation strategies in these professional groups. The results of this trial will be presented as soon as they are available.

## Abbreviations

BCA = British Chiropractic Association

CSP = Chartered Society of Physiotherapy

HC-PAIRS = Health Care Providers Pain and Impairment Relationship Scale

GCC = General Chiropractic Council

GOsC = General Osteopathic Council

LBP = Low back pain

NHS = National Health Service

RCT = Randomised controlled trial

RCGP = Royal College of General Practitioners

SPSS = Statistical Package for Social Scientists

WBS = Working Backs Scotland

## Competing interests

The author(s) declare that they have no competing interests.

## Authors' contributions

DE conceived, operationalised and co-ordinated the trial and drafted the manuscript. All authors participated in the design of the trial and have read and approved the final manuscript.

## Pre-publication history

The pre-publication history for this paper can be accessed here:



## References

[B1] Clinical Standards Advisory Group (CSAG) (1994). Clinical Standards Advisory Group Report on Back Pain.

[B2] Maniadakis N, Gray A (2000). The economic burden of back pain in the UK. Pain.

[B3] Breen AC (2003). The implementation of consensus guidelines. http://www.rygdoktor.dk/.

[B4] McGuirk B, King W, Govind J, Lowry J, Bogduk N (2001). Safety, efficacy, and cost effectiveness of evidence-based guidelines for the management of acute low back pain in primary care. Spine.

[B5] Rossignol M, Abenhaim L, Séguin P, Neveu A, Collet J, Ducruet T, Shapiro S (2000). Coordination of primary health care for back pain: A randomized controlled trial. Spine.

[B6] Field MJ, Lohr KN, Eds (1990). Clinical Practice Guidelines: Directions for a New Program.

[B7] Cherkin D, Deyo RA, Berg AO, Bergman JJ, Lishner DM (1991). Evaluation of a physician education intervention to improve primary care for low-back pain. I. Impact on physicians. Spine.

[B8] Deyo RA, Schall M, Berwick DM, Nolan T, Carver P (2000). Continuous quality improvement for patients with back pain. J Gen Intern Med.

[B9] Buchbinder R, Jolley D, Wyatt M (2001). Population based intervention to change back pain beliefs and disability: three part evaluation. BMJ.

[B10] Buchbinder R, Jolley D, Wyatt M (2001). 2001 Volvo award winner in clinical studies: effects of a media campaign on back pain beliefs and its potential influence on management of low back pain in general practice. Spine.

[B11] Underwood M, O'Meara S, Harvey E, The UK BEAM Trial Team (2002). The acceptability to primary care staff of a multidisciplinary training package on acute back pain guidelines. Fam Pract.

[B12] Bekkering GE, Engers AJ, Wensing M, Hendriks HJ, van Tulder MW, Oostendorp RA, Bouter LM (2003). Development of an implementation strategy for physiotherapy guidelines on low back pain. Aust J Physiother.

[B13] Bekkering GE, Hendriks HJM, Koes BW, Oostendorp RAB, Ostelo RWJG, Thomassen JMC, van Tulder MW (2003). Clinical guidelines on physiotherapy for low back pain. Physiotherapy.

[B14] Breen A, Carr E, Mann E, Crossen-White H (2004). Acute back pain management in primary care: a qualitative pilot study of the feasibility of a nurse-led service in general practice. J Nurs Manag.

[B15] Waddell G (2004). The Back Pain Revolution.

[B16] Burton AK, Waddell G (1998). Clinical guidelines in the management of low back pain. Baillieres Clin Rheumatol.

[B17] Koes BW, van Tulder MW, Ostelo R, Burton AK, Waddell G (2001). Clinical guidelines for the management of low back pain in primary care: an international comparison. Spine.

[B18] Staal JB, Hlobil H, van Tulder MW, Waddell G, Burton AK, Koes BW, van Mechelen W (2003). Occupational health guidelines for the management of low back pain: an international comparison. Occup Environ Med.

[B19] van Tulder MW, Tuut M, Pennick V, Bombardier C, Assendelft WJ (2004). Quality of primary care guidelines for acute low back pain. Spine.

[B20] Waddell G, McIntosh A, Hutchinson A, Feder G, Lewis M (1996). Clinical Guidelines for the Management of Acute Low Back Pain.

[B21] Waddell G, McIntosh A, Hutchinson A, Feder G, Lewis M (1999). Clinical Guidelines for the Management of Acute Low Back Pain.

[B22] Carter JT, Birrell LN, Eds (2000). Occupational health guidelines for the management of low back pain at work – principal recommendations.

[B23] Waddell G, Burton AK (2000). Occupational health guidelines for the management of low back pain at work – evidence review.

[B24] Harvey E, Burton AK, Klaber-Moffett J, Breen AC (2003). Spinal manipulation for low-back pain: a treatment package agreed by the UK chiropractic, osteopathy and physiotherapy professional associations. Man Ther.

[B25] Government Statistical Service (2000). The prevalence of back pain in Great Britain in 1998 Bulletin based on the Office for National Statistics (ONS) Omnibus Survey module conducted in March, April and June 1998.

[B26] Mason V (1994). The prevalence of low back pain in Great Britain A report on Office of Census & Population Surveys (OPCS) omnibus survey data produced on behalf of the Department of Health.

[B27] Funk SG, Champagne MT, Wiese RA, Tornquist EM (1991). BARRIERS: the barriers to research utilization scale. Appl Nurs Res.

[B28] Funk SG, Champagne MT, Wiese RA, Tornquist EM (1991). Barriers to using research findings in practice: the clinician's perspective. Appl Nurs Res.

[B29] Barta KM (1995). Information-seeking, research utilization, and barriers to research utilization of pediatric nurse educators. J Prof Nurs.

[B30] Dunn V, Crichton N, Roe B, Seers K, Williams K (1997). Using research for practice: a UK experience of the BARRIERS Scale. J Adv Nurs.

[B31] Metcalfe C, Lewin R, Wisher S, Perry S, Bannigan K, Klaber-Moffett J (2001). Barriers to implementing the evidence base in four NHS therapies. Physiotherapy.

[B32] Schers H, Wensing M, Huijsmans Z, van Tulder M, Grol R (2001). Implementation barriers for general practice guidelines on low back pain a qualitative study. Spine.

[B33] Gabbay J, le May A (2004). Evidence based guidelines or collectively constructed "mindlines?" Ethnographic study of knowledge management in primary care. BMJ.

[B34] Hibble A, Kanka D, Pencheon D, Pooles F (1998). Guidelines in general practice: the new Tower of Babel?. BMJ.

[B35] Breen AC (1977). Chiropractors and the treatment of back pain. Rheumatol Rehabil.

[B36] Burton AK (1981). Back pain in osteopathic practice. Rheumatol Rehabil.

[B37] Pringle M, Tyreman S (1993). Study of 500 patients attending an osteopathic practice. Br J Gen Pract.

[B38] Pedersen P (1994). A survey of chiropractic practice in Europe. Euro J Chiro.

[B39] Foster NE, Thompson KA, Baxter GD, Allen JM (1999). Management of nonspecific low back pain by physiotherapists in Britain and Ireland. A descriptive questionnaire of current clinical practice. Spine.

[B40] Gracey JH, McDonough SM, Baxter GD (2002). Physiotherapy management of low back pain: a survey of current practice in Northern Ireland. Spine.

[B41] Foster NE, Doughty G (2002). Using clinical guidelines and research evidence in the management of back pain patients: a qualitative study of musculoskeletal physiotherapists in Britain. Presented at the International Forum V for Primary Care Research on Low Back Pain, Montréal, Canada.

[B42] Rebain R, Baxter GD, McDonough S (2003). The passive straight leg raising test in the diagnosis and treatment of lumbar disc herniation: a survey of United kingdom osteopathic opinion and clinical practice. Spine.

[B43] Linton SJ, Vlaeyen J, Ostelo R (2002). The back pain beliefs of health care providers: are we fear-avoidant?. J Occup Rehabil.

[B44] Evans DW, Foster NE, Vogel S, Breen AC (2003). Implementing evidence-based practice in the UK physical therapy professions: do they want it and do they feel they need it?. Presented at the International Forum VI for Primary Care Research on Low Back Pain, Linkoping, Sweden.

[B45] Daykin AR, Richardson B (2004). Physiotherapists' pain beliefs and their influence on the management of patients with chronic low back pain. Spine.

[B46] Houben RMA, Vlaeyen JWS, Peters PMJC, Ostelo RWJG, Wolters PMJC, Stomp-van den Berg SGM (2004). Health care providers' attitudes and beliefs towards common low back pain: Factor structure and psychometric properties of the HC-PAIRS. Clin J Pain.

[B47] Houben RM, Gijsen A, Peterson J, de Jong PJ, Vlaeyen JW (2005). Do health care providers' attitudes towards back pain predict their treatment recommendations? Differential predictive validity of implicit and explicit attitude measures. Pain.

[B48] Houben RM, Ostelo RW, Vlaeyen JW, Wolters PM, Peters M, Stomp-van den Berg SG (2005). Health care providers' orientations towards common low back pain predict perceived harmfulness of physical activities and recommendations regarding return to normal activity. Eur J Pain.

[B49] Bishop A, Foster N (2005). Do physical therapists in the United Kingdom recognize psychosocial factors in patients with acute low back pain?. Spine.

[B50] Grol R, Thomas S, Roberts R (1995). Development and implementation of guidelines for family practice: lessons from The Netherlands. J Fam Pract.

[B51] Grol R, Zwaard A, Mokkink H, Dalhuijsen J, Casparie A (1998). Dissemination of guidelines: which sources do physicians use in order to be informed?. Int J Qual Health Care.

[B52] Grol R, Grimshaw J (1999). Evidence-based implementation of evidence-based medicine. Jt Comm J Qual Improv.

[B53] Grol R, Jones R (2000). Twenty years of implementation research. Fam Pract.

[B54] van Tulder MW, Croft PR, van Splunteren P, Miedema HS, Underwood MR, Hendriks HJM, Wyatt ME, Borkan JM (2002). Disseminating and implementing the results of back pain research in primary care. Spine.

[B55] NHS Centre for Reviews and Dissemination (1999). Getting evidence into practice. Effective Health Care.

[B56] Freemantle N (2000). Implementation strategies. Fam Pract.

[B57] Davis DA, Thomson MA, Oxman AD, Haynes RB (1995). Changing physician performance. A systematic review of the effect of continuing medical education strategies. J A M A.

[B58] Thorsen T, Mäkelä M, Eds (1999). Changing Professional Practice: Theory and Practice of Clinical Guidelines Implementation.

[B59] Freemantle N, Harvey EL, Wolf F, Grimshaw JM, Grilli R, Bero LA (2000). Printed educational materials: effects on professional practice and health care outcomes. Cochrane Database Syst Rev.

[B60] Grimshaw JM, Shirran L, Thomas R, Mowatt G, Fraser C, Bero L, Grilli R, Harvey E, Oxman A, O'Brien MA (2001). Changing provider behavior: an overview of systematic reviews of interventions. Med Care.

[B61] Grimshaw JM, Thomas RE, MacLennan G, Fraser C, Ramsay CR, Vale L, Whitty P, Eccles MP, Matowe L, Shirran L, Wensing M, Dijkstra R, Donaldson C (2004). Effectiveness and efficiency of guideline dissemination and implementation strategies. Health Technol Assess.

[B62] Oxman AD, Thomson MA, Davis DA, Haynes RB (1995). No magic bullets: a systematic review of 102 trials of interventions to improve professional practice. CMAJ.

[B63] Grimshaw J, Eccles M, Tetroe J (2004). Implementing clinical guidelines: current evidence and future implications. J Contin Educ Health Prof.

[B64] Cohen SJ, Weinberger M, Hui SL, Tierney WM, McDonald CJ (1985). The impact of reading on physicians' nonadherence to recommended standards of medical care. Soc Sci Med.

[B65] Emslie C, Grimshaw J, Templeton A (1993). Do clinical guidelines improve general practice management and referral of infertile couples?. B M J.

[B66] Bearcroft PW, Small JH, Flower CD (1994). Chest radiography guidelines for general practitioners: a practical approach. Clin Radiol.

[B67] Grimshaw J (1998). Evaluation of four quality assurance initiatives to improve out-patient referrals from general practice to hospital. Dissertation University of Aberdeen.

[B68] Mesters I, Meertens R, Kok G, Parcel GS (1994). Effectiveness of a multidisciplinary education protocol in children with asthma (0–4 years) in primary health care. J Asthma.

[B69] Oakeshott P, Kerry SM, Williams JE (1994). Randomised controlled trial of the effect of the Royal College of Radiologists' guidelines on general practitioners' referral for radiographic examination. Br J Gen Pract.

[B70] Rabin DL, Boekeloo BO, Marx ES, Bowman MA, Russell NK, Willis AG (1994). Improving office-based physician's prevention practices for sexually transmitted diseases. Ann Intern Med.

[B71] Bland JM (2000). Sample size in guidelines trials. Fam Pract.

[B72] Wood J, Freemantle N (1999). Choosing an appropriate unit of analysis in trials of interventions that attempt to influence practice. J Health Serv Res Policy.

[B73] O'Brien K, Wright J, Conboy F, Bagley L, Lewis D, Read M, Thompson R, Bogues W, Lentin S, Parr G, Aron B (2000). The effect of orthodontic referral guidelines: a randomised controlled trial. Br Dent J.

[B74] Bland JM (2004). Cluster randomised trials in the medical literature: two bibliometric surveys. BMC Med Res Methodol.

[B75] Eldridge SM, Ashby D, Feder GS, Rudnicka AR, Ukoumunne OC (2004). Lessons for cluster randomized trials in the twenty-first century: a systematic review of trials in primary care. Clinical Trials.

[B76] Hahn S, Puffer S, Torgerson DJ, Watson J (2005). Methodological bias in cluster randomised trials. BMC Med Res Methodol.

[B77] Farrin A, Russel I, Torgerson D, Underwood M (2005). Differential recruitment in a cluster randomised trial in primary care – the experience of the UK Back pain, Exercise, Active management and Manipulation (UK BEAM) feasibility study. Clinical Trials.

[B78] Bekkering GE, van Tulder MW, Hendriks EJM, Koopmanschap MA, Knol DL, Bouter LM, Oostendorp RAB (2005). Implementation of clinical guidelines on physical therapy for patients with low back pain: randomized trial comparing patient outcomes after a standard and active implementation strategy. Phys Ther.

[B79] Bekkering GE, Hendriks HJ, van Tulder MW, Knol DL, Hoeijenbos M, Oostendorp RA, Bouter LM (2005). Effect on the process of care of an active strategy to implement clinical guidelines on physiotherapy for low back pain: a cluster randomised controlled trial. Qual Saf Health Care.

[B80] Engers AJ, Wensing M, van Tulder MW, Timmermans A, Oostendorp RA, Koes BW, Grol R (2005). Implementation of the Dutch low back pain guideline for general practitioners: a cluster randomized controlled trial. Spine.

[B81] Schectman JM, Schroth WS, Verme D, Voss JD (2003). Randomized controlled trial of education and feedback for implementation of guidelines for acute low back pain. J Gen Intern Med.

[B82] Dey P, Simpson CW, Collins SI, Hodgson G, Dowrick CF, Simison AJ, Rose MJ (2004). Implementation of RCGP guidelines for acute low back pain: a cluster randomised controlled trial. Br J Gen Pract.

[B83] Sculpher M (2000). Evaluating the cost-effectiveness of interventions designed to increase the utilization of evidence-based guidelines. Fam Pract.

[B84] Suarez-Almazor ME, Russell AS (1998). The art versus the science of medicine. Are clinical practice guidelines the answer?. Ann Rheum Dis.

[B85] Harding S (1997). McTimoney Chiropractic: the first 25 years.

[B86] UK BEAM Trial Team (2003). UK Back pain Exercise And Manipulation (UK BEAM) trial – national randomised trial of physical treatments for back pain in primary care: objectives, design and interventions. BMC Health Serv Res.

[B87] Working Backs Scotland Online. http://www.workingbacksscotland.com.

[B88] Buchbinder R, Jolley D (2004). Population based intervention to change back pain beliefs: three year follow up population survey. B M J.

[B89] Feder G, Grange W, Hamlyn P, Hull S, Hussaini A, Kidd B, Kidner S, Montgomery J (1997). Clinical guidelines for the management of acute low back pain in East London primary care.

[B90] Stevenson K, Hay EM (2001). A survey of GP's and physiotherapists (PT) views on a physiotherapy led back pain service. Presented at the Society for Back Pain Research, Bristol, UK.

[B91] Silagy CA, Weller DP, Lapsley H, Middleton P, Shelby-James T, Fazekas B (2002). The effectiveness of local adaptation of nationally produced clinical practice guidelines. Fam Pract.

[B92] Deane K (2004). Development of guidelines on lower back pain. Presented at the Chartered Society of Physiotherapists Annual Congress, Birmingham, UK.

[B93] Kendall N, Linton SJ, Main CJ (1997). Guide to Assessing Psychological Yellow Flags in Acute Low Back Pain: Risk factors for long-term disability and work loss.

[B94] Roland M, Burton K, Waddell G, Klaber-Moffett J, Main C (1996). The Back Book.

[B95] Burton AK, Waddell G, Tillotson KM, Summerton N (1999). Information and advice to patients with back pain can have a positive effect. A randomized controlled trial of a novel educational booklet in primary care. Spine.

[B96] Health and Safety Executive (2000). Back in work: managing back pain in the workplace A leaflet for employers and workers in small businesses.

[B97] Grol R, Dalhuijsen J, Thomas S, Veld C, Rutten G, Mokkink H (1998). Attributes of clinical guidelines that influence use of guidelines in general practice: observational study. B M J.

[B98] Michie S, Johnston M (2004). Changing clinical behaviour by making guidelines specific. B M J.

[B99] Hayward RS, Guyatt GH, Moore KA, McKibbon KA, Carter AO (1997). Canadian physicians' attitudes about and preferences regarding clinical practice guidelines. Can Med Assoc J.

[B100] Wennberg DE, Dickens JD, Biener L, Fowler FJ, Soule DN, Keller RB (1997). Do physicians do what they say? The inclination to test and its association with coronary angiography rates. J Gen Intern Med.

[B101] Hartley RM, Charlton JR, Jarman B, Harris CM (1985). Case history questionnaires in the study of doctors' use of resources: Are they measuring what we want?. Med Care.

[B102] Jones TV, Gerrity MS, Earp J (1990). Written case simulations: Do they predict physicians' behavior?. J Clin Epidemiol.

[B103] Morrell DC, Roland MO (1990). Analysis of referral behaviour: responses to simulated case histories may not reflect real clinical behaviour. Br J Gen Pract.

[B104] Peabody JW, Luck J, Glassman P, Dresselhaus TR, Lee M (1997). Comparison of vignettes, standardized patients, and chart abstraction. A prospective study of 3 methods for measuring quality. J Am Med Assoc.

[B105] Bombardier C, Jansz G, Maetzel A (1995). Primary care physicians' knowledge, confidence, and attitude in the management of acute low back pain (ALBP). Arthritis Rheum.

[B106] Ammendolia C, Bombardier C, Hogg-Johnson S, Glazier R (2002). Views on radiography use for patients with acute low back pain in an Ontario community. J Manip Physiol Ther.

[B107] Li LC, Bombardier C (1999). Physical therapy management of low back pain: an exploratory survey of therapist approaches. Phys Ther.

[B108] Maetzel A, Johnson SH, Woodbury M, Bombardier C (2000). Use of grade membership analysis to profile the practice styles of individual physicians in the management of acute low back pain. J Clin Epidemiol.

[B109] Rainville J, Carlson N, Polatin P, Gatchel RJ, Indahl A (2000). Exploration of physicians' recommendations for activities in chronic low back pain. Spine.

[B110] Rainville J, Bagnall D, Phalen L (1995). Health care providers' attitudes and beliefs about functional impairments and chronic back pain. Clin J Pain.

[B111] Riley JF, Ahern DK, Follick MJ (1988). Chronic back pain and functional impairment: assessing beliefs about their relationship. Arch Phys Med Rehab.

[B112] Barrios FX, Riley JF (1987). Cognitive factors and disability in chronic pain: is there more than just depression?. Presented at the 21st Annual Meeting for the Advancement of Behavioural Therapy, Boston.

[B113] Put CL, Witkower A (1991). Pain and impairment beliefs in patients treated in an interdisciplinary inpatient pain program. Presented at the American Pain Society 10th Annual Meeting, New Orleans, LA.

[B114] Slater MA, Hall HF, Atkinson JH, Garfin SR (1991). Pain and impairment beliefs in chronic low back pain; validation of the Pain and Impairment Relationship Scale (PAIRS). Pain.

[B115] Russell ML, Verhoef MJ, Injeyan HS, McMorland DG (2004). Response rates for surveys of chiropractors. J Manip Physiol Ther.

[B116] Konstantinou K, Foster N, Rushton A, Baxter D (2002). The use and reported effects of mobilization with movement techniques in low back pain management; a cross-sectional descriptive survey of physiotherapists in Britain. Man Ther.

[B117] Bush T, Cherkin D, Barlow W (1993). The impact of physician attitudes on patient satisfaction with care for low back pain. Arch Fam Med.

[B118] Smucker DR, Konrad TR, Curtis P, Carey TS (1998). Practitioner self-confidence and patient outcomes in acute low back pain. Arch Fam Med.

[B119] Connolly BH, Lupinnaci NS, Bush AJ (2001). Changes in attitudes and perceptions about research in physical therapy among professional physical therapist students and new graduates. Phys Ther.

